# An integrated phenotypic and genomic approach to characterize MBL-producing Enterobacterales strains circulating in a Sicilian transplant center

**DOI:** 10.1128/spectrum.02077-25

**Published:** 2025-11-26

**Authors:** Claudia Vaiana, Carol Lee, Roberta Vazzana, Andrea Cona, Giovanni Mulè, Caterina Amato, Francesco Monaco, Daniele Di Carlo, Pier Giulio Conaldi, Valentina Agnese, Nicola Cuscino, Alessandra Mularoni, Alessia Gallo

**Affiliations:** 1 Department of Research, IRCCS ISMETT (Istituto Mediterraneo per i Trapianti e Terapie ad Alta Specializzazione) 18326, Palermo, Italy; 2 Hospital das Clínicas, Universidade de São Paulo 28133, São Paulo, Brazil; 3 Unit of Infectious Diseases, IRCCS ISMETT (Istituto Mediterraneo per i Trapianti e Terapie ad Alta Specializzazione) 18326, Palermo, Italy; 4 Laboratory of Clinical Pathology, Microbiology and Virology, IRCCS ISMETT (Istituto Mediterraneo per i Trapianti e Terapie ad Alta Specializzazione) 18326, Palermo, Italy; University of Guelph College of Biological Science, Guelph, Canada

**Keywords:** MBL, whole genome sequencing, antimicrobial stewardship, antimicrobial resistance, difficult-to-treat infections, *K. pneumoniae*, *E. coli*

## Abstract

Metallo-β-lactamases (MBLs)-producing Enterobacterales infections represent a serious threat in clinical practice due to high mortality rates associated and the limited therapeutic options available. Given their increased dissemination across Europe and their involvement in hospital outbreaks, further epidemiological investigations are required. Here, we provide a comprehensive characterization, by integrating clinical, genomic, and phenotypic data, of 22 MBL-carrying Enterobacterales strains (17 *Klebsiella pneumoniae* and 5 *Escherichia coli*), isolated from critically ill patients admitted to IRCCS ISMETT from 2021 to 2024. Bacterial antimicrobial susceptibility was evaluated using phenotypic methods. Using a whole-genome sequencing approach, we identified the sequence types (STs) and depicted the resistome and virulome content. This study revealed the circulation of alarming MBL-producing strains in our hospital, with New Delhi MBL (82%), followed by Verona integron-encoded MBL (VIM) (18%). Beyond carbapenem resistance, these strains exhibited high resistance rates to crucial antibiotics, including amikacin (63%), in addition to reduced susceptibility to cefiderocol (50%). Moreover, the emergence of VIM-1 *K. pneumoniae* isolates resistant to colistin was observed. Genomic analysis revealed the presence of high-risk clones such as *K. pneumoniae* ST147 and ST512 and *E. coli* ST648, necessitating vigilant monitoring. The resistome analysis showed widespread co-occurrence of several resistance genes, contributing to the multidrug-resistant (MDR) phenotype. The convergence of resistance and virulence characteristics highlights the urgent need for integrated genomic surveillance and improved measures to control the spread of hypervirulent MDR bacteria.

**IMPORTANCE:**

The increasing prevalence of metallo-β-lactamase-producing Enterobacterales is a significant healthcare concern. Traditional phenotypic methods, commonly employed in diagnostic labs, are essential for the resistance marker detection and antimicrobial susceptibility testing. Instead, bacterial genetic features, including additional resistance genes and virulence determinants that have significant clinical implications, remain neglected. To overcome this critical gap, an integrated approach, consisting of phenotypic and genomic analyses, is crucial for gaining essential insights that guide both comprehensive patient management and effective epidemiological surveillance. Our findings highlight the importance of antimicrobial stewardship, antimicrobial susceptibility testing, and control measures to limit the spread of high-risk multidrug-resistant bacteria.

## INTRODUCTION

Carbapenem-resistant Enterobacterales (CRE) constitute a major global health concern due to their rapid dissemination in healthcare settings and their ability to cause severe infections, which are associated with substantial morbidity and mortality (1). The primary mechanism driving carbapenem resistance in CRE is the production of β-lactamases (2). Among these, metallo-β-lactamases (MBLs) are of particular concern, with New Delhi MBL (NDM), Verona integron-encoded MBL (VIM), and imipenemase (IMP) being their most epidemiologically important members (3). MBLs are endemic in India and Southeast Asia, with the largest burden of cases (4). However, in recent years, clinical events involving these enzymes have been increasingly reported worldwide, especially in Europe (4, 5). This growing prevalence is alarming due to their ability to hydrolyze nearly all β-lactams (BLs), including penicillins, cephalosporins, carbapenems, and novel BL/β-lactamase inhibitor (BLI) combinations, and their frequent association with other resistance mechanisms, including co-production of other β-lactamases, resulting in a highly multidrug-resistant (MDR) phenotype (6, 7). In Italy, VIM has historically been the most prevalent MBL among Enterobacterales and also the predominant carbapenemase in *Pseudomonas aeruginosa* (4). However, a significant outbreak of NDM-producing *Klebsiella pneumoniae* ST147 in Tuscany may signal a shift in this distribution (8). Since this event, an increasing number of NDM-producing isolates have been reported across other Italian regions (9, 10), raising concerns about the further spread of this resistance mechanism. Compounding this issue, some strains showed reduced susceptibility to cefiderocol, a critical last-line agent for these infections (11). The acquisition of MBLs is strongly associated with factors such as prior antimicrobial exposure, intensive care unit (ICU) admission, prolonged hospitalization, comorbidities, and invasive devices (12). Importantly, previous colonization significantly increases the risk of subsequent infection (13), as demonstrated by the higher probability of bloodstream infection development in rectal carriers of NDM-producing *K. pneumoniae* than in those colonized with KPC-producing strains (14), an event most likely linked to the specific clonal lineage rather than the carbapenemase type. These findings underscore the importance of active surveillance and infection control measures to limit intra-hospital horizontal transmission (15). Mortality associated with MBL-producing Enterobacterales infections is high, ranging from 21% to 44%, and up to 55% attributable (16). Emerging data indicate a strong correlation between the selected antibiotic therapy and patient outcomes. The combination of ceftazidime-avibactam and aztreonam is currently regarded as the preferred treatment for MBL-producing Enterobacterales, showing superior efficacy compared to alternative regimens (17). The expanding distribution of MBL-harboring Enterobacterales and the limited treatment arsenal highlight the urgent need for robust infection control and optimized therapeutic strategies (18). In this context, our study aimed to comprehensively analyze MBL-producing Enterobacterales isolated from critically ill patients. We emphasized the genomic characterization of these strains as a pivotal tool to (i) identify the genetic determinants of antimicrobial resistance and virulence, (ii) define circulating sequence types (STs), and (iii) assess clonal relationships and identify potential transmission clusters. To the best of our knowledge, this is the first study that integrates patients’ clinical data with genomic profiling of MBL-producing organisms using this approach.

## MATERIALS AND METHODS

### Patient data collection and bacterial strains isolation

During the period from January 2021 to September 2024, 127 patients admitted at IRCCS ISMETT, a solid organ transplant center, located in Palermo (Italy), were found to have positive cultures for MBL-producing Enterobacterales. Among them, 101/127 patients were colonized without signs of infection, while 26/127 patients developed infection. Of these, only four patients were excluded from this study because clinical data or bacterial isolates were missing. Patients were followed up until death or 30 days from infection. The last follow-up date was 20 April 2025, used as the censoring date for patients still alive.

We retrospectively collected patient demographic and clinical data, including age, sex, admission and discharge dates, underlying conditions, antibiotic treatments, and clinical outcomes from the hospital electronic records. Five of these isolates were collected from clinically relevant samples, three in blood, one in bile fluid, and one in the respiratory tract, and were directly linked to infectious episodes. The remaining 17 isolates, initially identified during colonization, were linked to subsequent infections using the concordance criteria, established by Falcone et al. (14). Based on these criteria, we confidently assumed that these colonization-related isolates, showing identical resistance markers and antimicrobial profiles, were responsible for the subsequent infectious events. Bacterial identification and antimicrobial susceptibility tests were performed by the hospital diagnostic laboratory as part of the routine management of the patients. Subsequently, these isolates were cryopreserved at −80°C in Luria-Bertani broth with 20% glycerol for future analyses, including additional antibiotic susceptibility testing and genomic investigations.

### Bacterial identification, antimicrobial susceptibility testing, and MBL detection

Bacterial identification was performed by matrix-assisted laser desorption ionization–time-of-flight mass spectrometry Vitek MS system (bioMérieux, Marcy-l'Étoile, France). Antimicrobial susceptibility to most of the antibiotics of interest was investigated using the VITEK 2 system (bioMérieux, Marcy-l'Étoile, France), while resistance to colistin was confirmed using Sensititre (Thermo Fisher Scientific, MA, USA). Susceptibility to cefiderocol was tested with the ComASP cefiderocol microdilution panel (0.008 µg/mL–128 µg/mL) (Liofilchem, Roseto degli Abruzzi, Italy). Minimum inhibitory concentration (MIC) results were interpreted according to the EUCAST clinical breakpoints (EUCAST clinical breakpoint guidance v.15.0). Susceptibility to aztreonam-avibactam was tested using the ETEST Aztreonam/Avibactam (AZA) (0.016/4 µg/mL–256/4 µg/mL) (bioMérieux, Marcy-l'Étoile, France). Production of class A and B carbapenemases was verified using carbapenemase inhibition tests with boronic acid derivates and dipicolinic acid/EDTA (Rosco Diagnostica A/S, Taastrup, Denmark). Identification of the five major carbapenemases (KPC, NDM, VIM, IMP, and OXA-48-like) from bacterial isolates was performed using immunochromatographic tests, including NG-Test Carba 5 (NG Biotech, Guipry, France) or the lateral flow immunoassay (Medomics), according to the manufacturer’s instructions.

### DNA extraction and whole-genome sequencing (WGS)

Pure bacterial colonies were grown on MacConkey agar plates, and then genomic DNA was extracted using the QIAamp DNA Mini Kit (Qiagen, Hilden, Germany), by following the manufacturer’s instructions. DNA was subsequently quantified using the Qubit 2.0 fluorometer (Invitrogen, Carlsbad, CA, USA) with the Qubit dsDNA Broad Range Assay Kit. DNA library preparation was performed using the Illumina DNA Prep Kit (Illumina, San Diego, CA, USA) according to the manufacturer’s instructions. Nextera DNA CD Indexes (96 Indexes–96 Samples; Illumina, San Diego, CA, USA) were ligated after amplification. DNA libraries were evaluated on the Agilent 4200 TapeStation System (Agilent Technologies Ltd., USA), while library concentration was checked using the Qubit 2.0 fluorometer (Invitrogen, MA, USA). Whole-genome sequencing was performed according to the manufacturer’s instructions on the NextSeq 550 System using a 2 × 150 bp paired-end library (Illumina Inc., San Diego, CA, USA).

### Bioinformatic analysis

The draft genome and plasmids were assembled using SPAdes v.3.15.12 software (https://github.com/ablab/spades) (19), and automatic annotation was performed with Prokka v.1.14.5 (https://github.com/tseemann/prokka) (20). The MLST STs were identified on the MLST database hosted by the CGE (https://cge.food.dtu.dk/services/MLST/) (21). Subsequently, the Kaptive Web algorithm (22) and SerotypeFinder 2.0 (23) were employed for *in silico* serotyping of *K. pneumoniae* and *Escherichia coli* isolates, respectively. Antimicrobial resistance genes (ARGs) and virulence factors were detected using CARD (24) and VFDB (25), respectively. *K. pneumoniae* strain cohort was additionally assessed for their virulence score using Kleborate (26). Plasmid incompatibility types were identified using PlasmidFinder on the CGE (https://cge.food.dtu.dk/services/PlasmidFinder/) website (27). Contigs containing MBL genes were initially aligned to plasmids identified using PlasmidFinder with the BLASTn algorithm (BLAST+ v.2.16.0;) (28). Contigs showing less than 80% identity and coverage with the reference plasmids were subsequently re-aligned against the NCBI nt database for further characterization. Kpn1 and Ecoli23 isolates were used as reference genomes for the core-genome SNP (cgSNP) analysis. The number of SNPs in the core genome was calculated from the *de novo* assembled genomes for isolates with the same ST (ST147, 512, 648, 225) using the Harvest software suite (Parsnp), including a cgSNP-based phylogenetic tree. A minimum spanning tree was constructed using R software (v.4.2.2) (https://www.R-project.org/).

## RESULTS

### Clinical characteristics and cases’ description

During the study period, a total of 22 patients with infection due to MBL-producing organisms were included. The median age of the study population was 61.5 years (IQR, 48.8–66.8 years), and the majority were male (54.5%). Pre-existing comorbidities were frequent, with cardiovascular disease (54.5%), chronic liver disease (27.7%), and chronic kidney disease (22.7%) being the most prevalent. Patients’ characteristics of the study cohort are detailed in Table 1. Half of the patients (50%) were solid organ transplant recipients: liver (36.3%) and kidney (18.1%) transplants being the most common. Seventeen patients (77.2%) had been hospitalized for more than 2 days in the previous 3 months, and half of the included patients had been transferred from another healthcare facility. Prior antibiotic use within 90 days before isolation of the MBL-producing organism, whether from surveillance or clinical specimens, was reported in 72.7% (16/22) of patients. Among those, BL/BLI combinations were the most frequently used agents (68.7%), followed by carbapenems (25%), third-generation cephalosporins and colistin (each 18.7%), quinolones, and cefiderocol (each 12.5%). The median length of hospital stay (LOS) was 48 days (IQR, 27–104 days). Co-colonization with KPC-producing organisms was observed in 68.1% (15/22) of patients, with 33.3% (5/15) of these harboring isolates co-producing both KPC and MBL enzymes.

**TABLE 1:** Characteristics of the 22 patients hospitalized with infection due to MBL-producing Enterobacterales*^a^*

Patients’ characteristic	Infection (*N* = 22)
Age	55 (2–80 years)
Male sex	12 (54.5%)
ICU stay (>2 days)	18 (81.8%)
Comorbid conditions	
Cardiovascular disease	12 (54.5%)
Diabetes mellitus	3 (13.6%)
Respiratory tract disease	2 (9%)
Chronic kidney disease	5 (22.7%)
Chronic liver disease	6 (27.7%)
Malignancy	4 (18.1%)
Organ solid transplant	11 (50%)
Liver	4 (36.3%)
Kidney	2 (18.1%)
Lung	2 (18.1%)
Heart	2 (18.1%)
Kidney and liver	1 (9%)
RT or CT last 90 days	1 (4.5%)
Immunosuppression	11 (50%)
Previous hospitalization in the last 3 months	17 (77.2%)
Transfer patient (>2 days institutionalized in another facility)	11 (50%)
Use of antibiotic in the last 90 days	16 (72.7%)
Carbapenem	4 (25%)
Third-generation cephalosporin	3 (18.7%)
BL/BLI combinations^*b*^	11 (68.7%)
Colistin	3 (18.7%)
Quinolones	2 (12.5%)
Cefiderocol	2 (12.5%)
LOS	48 days (5–232 days)
Invasive devices	19 (76.4%)
Abdominal surgery	6 (31.5%)
Cardiac surgery	4 (21%)
Thoracic surgery	2 (10.5%)
Endoscopic procedure	7 (36.8%)
Use of invasive diapositives (>2 days)^*c*^	20 (90.9%)
Co-colonization with KPC enzyme	15 (68.1%)
Co-producer of KPC and MBL	5 (33.3%)

^*a*^ CT, chemotherapy; RT, radiotherapy.

^*b*^ Corresponds to piperacillin-tazobactam, ceftolozane-tazobactam, and ceftazidime-avibactam.

^*c*^ Including central venous catheter, urinary catheter, endotracheal intubation, tracheostomy, hemodialysis, and other devices (surgical drains).

The most common sources of infection were bloodstream (45.4%) and intra-abdominal site (22.7%). Clinical infection characteristics and treatment data are summarized in Table 2. Among the 10 bloodstream infections, only two were caused by *E. coli*, harboring NDM-5, and the remaining eight were caused by *K. pneumoniae*. Interestingly, 27.2% of patients (6/22) developed a subsequent infection at a different anatomical site during follow-up; notably, only one case involved the same anatomical site as the initial infection. The primary treatment regimen across the cohort was the combination of ceftazidime-avibactam and aztreonam, used in 54.4% (12/22) of cases, followed by cefiderocol in 18.1% (4/22) and colistin in 13.6% (3/22). Among patients with bloodstream infections, ceftazidime-avibactam plus aztreonam was the main regimen in 60% (6/10), while cefiderocol was used in 30% of cases (3/10). For intra-abdominal infections, a combination of tigecycline and an aminoglycoside played a role in 40% (2/5) of the cases. Among patients analyzed, the 30-day mortality rate was 31.8% (7/22). At the last follow-up in March 2025, 12 patients (54.5%) were alive, 7 (31.8%) had died, and for 3 (13.6%), follow-up information was not available. Of the seven deceased patients, five died during hospitalization, with MBL infection identified as the primary cause in three cases. The remaining two deaths occurred during the study follow-up and were attributed to causes unrelated to the MBL infection.

**TABLE 2:** Characteristics of MBL-associated infections and clinical management^*a*^

Year	Sex	Age	Length of stay (no. days)	Isolate	Species	Carbapenemases	Source of infection	Treatment	Subsequent infection/Site	30-day mortality
2021	M	74	49	Ecoli15	*E. coli*	NDM-5	Abdominal	AMK, TGC, FOS	Yes/Abdominal	Alive
2021	F	67	18	Ecoli16	*E. coli*	NDM-5	Abdominal	AMK, TGC, FOS	No	Alive
2021	M	59	99	Ecoli17	*E. coli*	NDM-5	Blood	CZA + ATM, AMK	Yes/Abdominal	Alive
2022	F	2	5	Ecoli22	*E. coli*	VIM-1	Abdominal	CZA + ATM, CST	No	Alive
2022	M	62	232	Kpn19	*K. pneumoniae*	VIM-1	Blood	CZA + ATM	No	Deceased
2022	F	70	106	Kpn21	*K. pneumoniae*	VIM-1	Blood	CZA + ATM	No	Deceased
2022	M	46	47	Kpn18	*K. pneumoniae*	VIM-1	Blood	FDC	No	Transferred^*b*^
2023	M	48	87	Kpn6	*K. pneumoniae*	NDM-1	Abdominal	CZA + ATM	No	Deceased
2023	M	54	25	Ecoli23	*E. coli*	NDM-5	Blood	CZA + ATM	No	Deceased of infection
2023	M	69	123	Kpn7	*K. pneumoniae*	NDM-1	Respiratory	CST	No	Transferred^*b*^
2023	F	38	27	Kpn24	*K. pneumoniae*	NDM-1	Skin and soft tissue	CZA + ATM	No	Alive
2023	F	67	171	Kpn8	*K. pneumoniae*	NDM-1	Urinary	FOS	Yes/Respiratory	Deceased of infection
2024	F	63	8	Kpn1	*K. pneumoniae*	NDM-1	Abdominal	CZA + ATM	No	Alive
2024	M	66	35	Kpn3	*K. pneumoniae*	NDM-1	Blood	FDC	Yes/Urinary	Alive
2024	M	51	7	Kpn5	*K. pneumoniae*	NDM-1	Blood	CZA + ATM	Yes/Abdominal	Alive
2024	F	80	83	Kpn4	*K. pneumoniae*	NDM-1, KPC-3	Blood	CZA + ATM	No	Deceased
2024	M	62	131	Kpn2	*K. pneumoniae*	NDM-1	Blood	FDC	Yes/Abdominal	Deceased of infection
2024	M	61	31	Kpn9	*K. pneumoniae*	NDM-1	Blood	CST, CZA	No	Transferred^*b*^
2024	F	21	28	Kpn13	*K. pneumoniae*	NDM-1, KPC-3	Respiratory	FDC	No	Transferred^*b*^
2024	M	61	142	Kpn10	*K. pneumoniae*	NDM-1, KPC-3	Skin and soft tissue	CZA + ATM, AMK	No	Alive
2024	F	62	36	Kpn12	*K. pneumoniae*	NDM-1	Skin and soft tissue	N/A	No	Transferred^*b*^
2024	M	45	55	Kpn11	*K. pneumoniae*	NDM-1, KPC-3	Urinary	CZA + ATM	No	Alive

^*a*^ AMK, amikacin; ATM, aztreonam; CZA, ceftazidime-avibactam; CST, colistin; TGC, tigecycline; FDC, cefiderocol; FOS, fosfomycin; N/A, not available: the patient was transferred to another hospital; thus, subsequent treatment data were not available.

^*b*^ Transferred to another institution.

### Identification, antimicrobial susceptibility profile, and MBL detection

The study population included 17 *K*. *pneumoniae* and 5 *E. coli* isolates. The most prevalent MBL was NDM, detected in 82% of the isolates (18/22), and followed by VIM, present in 18% of the isolates (4/22). Among the NDM-positive isolates, 14 out of 18 carried the NDM-1 variant, while 4 out of 18 had NDM-5, which was exclusively found in *E. coli*. In contrast, just one VIM variant, VIM-1, was detected.

All isolates were examined for susceptibility to multiple antimicrobial agents, with particular focus on those frequently used for treatment of MBL-specific infections. As depicted in Table 3, consistent resistance was noted, as expected, across all isolates to fluoroquinolones (ciprofloxacin), last-generation cephalosporins (cefepime, ceftriaxone, and ceftazidime), and BL/BLI combinations (ceftolozane-tazobactam, ceftazidime-avibactam, and imipenem-relebactam), while susceptibility to meropenem-vaborbactam was observed only in 1 out of the 22 isolates. No isolate was susceptible to meropenem. Thus, isolates showed a typical antimicrobial resistance profile of MBL-producing bacteria. Nevertheless, almost all isolates (21/22) were also resistant to aztreonam, likely due to the presence of other β-lactamases not detected with phenotypic tests. Regarding aminoglycosides, all tested isolates were resistant to tobramycin, while 63.6% (14 out of 22) and 59% (13 out of 22) of isolates were resistant also to amikacin and gentamicin, respectively. The highest susceptibility rates were observed for colistin and cefiderocol, with 19/22 (86%) and 21/22 (95%) susceptible isolates, respectively. Interestingly, all isolates were susceptible to aztreonam-avibactam combination (Table 3).

**TABLE 3:** Sequence type (ST) and antimicrobial susceptibility profile of the isolates analyzed^*a*^^,^^*b*^

Isolate	Collection date (day/mo/yr)	Specimen source	Carbapenemases^*d*^	ST	Predicted serotype	MIC (µg/mL)^*c*^																		
						AMK	FEP	CTX	CAZ	CZA	C/T	CIP	CST^*e*^	GEN	IMR	MEM	MEV	TZP	TMP/SMX	TOB	ATM	TGC^*g*^	FDC^*f*^	AZA
Ecoli15^*h*^	16/09/2021	Biliary liquid	NDM-5	648	H4-O8	4 (S)	8 (R)	32 (R)	>32 (R)	>8 (R)	>16 (R)	>2 (R)	0.5 (S)	>8 (R)	>8 (R)	>8 (R)	>32 (R)	>64 (R)	≤20 (S)	>8 (R)	>32 (R)	≤0.5	2 (S)	1.5 (S)
Ecoli16	20/09/2021	Rectal swab	NDM-5	648	H4-O8	4 (S)	>16 (R)	>32 (R)	>32 (R)	>8 (R)	>16 (R)	>2 (R)	0.5 (S)	>8 (R)	>8 (R)	>8 (R)	32 (R)	>64 (R)	≤20 (S)	>8 (R)	>32 (R)	≤0.5	2 (S)	1.5 (S)
Ecoli17	12/10/2021	Rectal swab	NDM-5	648	H4-O8	8 (S)	>16 (R)	>32 (R)	>32 (R)	>8 (R)	>16 (R)	>2 (R)	0.5 (S)	>8 (R)	>8 (R)	>8 (R)	>32 (R)	>64 (R)	≤20 (S)	>8 (R)	>32 (R)	≤0.5	2 (S)	1.5 (S)
Ecoli22	10/10/2022	Rectal swab	VIM-1	1485	H42-O83	4 (S)	16 (R)	32 (R)	>32 (R)	>8 (R)	>16 (R)	>2 (R)	≤0.5 (S)	≤1 (S)	4 (R)	2 (I)	2 (S)	>64 (R)	>160 (R)	8 (R)	≤1 (S)	≤0.5	0.25 (S)	0.19 (S)
Ecoli23	30/04/2023	Rectal swab	NDM-5	405	H6-O102	>32 (R)	>16 (R)	>32 (R)	>32 (R)	>8 (R)	>16 (R)	>2 (R)	0.5 (S)	>8 (R)	>8 (R)	>8 (R)	32 (R)	>64 (R)	>160 (R)	>8 (R)	>32 (R)	≤0.5	2 (S)	3 (S)
Kpn18	23/01/2022	Rectal swab	VIM-1	225	K3-O2afg	≤1 (S)	>16 (R)	>32 (R)	>32 (R)	>8 (R)	>16 (R)	>2 (R)	>8 (R)	ND	4 (R)	>8 (R)	32 (R)	>64 (R)	>160 (R)	8 (R)	>32 (R)	>4	1 (S)	0.25 (S)
Kpn19	17/03/2022	Blood	VIM-1	392	K27-O4	8 (I)	>16 (R)	>32 (R)	>32 (R)	>8 (R)	>16 (R)	>2 (R)	>8 (R)	>8 (R)	>8 (R)	>8 (R)	>32 (R)	>64 (R)	>160 (R)	>8 (R)	>32 (R)	1	0.5 (S)	0.25 (S)
Kpn21	23/04/2022	Rectal swab	VIM-1	225	K3-O2afg	≤1 (S)	>16 (R)	>32 (R)	>32 (R)	>8 (R)	>16 (R)	>2 (R)	>8 (R)	2 (S)	>8 (R)	>8 (R)	32 (R)	>64 (R)	>160 (R)	8 (R)	>32 (R)	>4	1 (S)	0.25 (S)
Kpn24	05/08/2023	Rectal swab	NDM-1	147	K64-O2a	32 (R)	>16 (R)	>32 (R)	>32 (R)	>8 (R)	>16 (R)	>2 (R)	0.5 (S)	≤1 (S)	>8 (R)	>8 (R)	32 (R)	>64 (R)	80 (I)	>8 (R)	>32 (R)	4	0.5 (S)	0.125 (S)
Kpn6	18/11/2023	JP fluid	NDM-1	147	K64-O2a	16 (R)	>16 (R)	>32 (R)	>32 (R)	>8 (R)	>16 (R)	>2 (R)	0.5 (S)	≤1 (S)	>8 (R)	>8 (R)	>32 (R)	>64 (R)	>160 (R)	ND	>32 (R)	4	1 (S)	0.25 (S)
Kpn7	26/11/2023	Tracheal aspirate	NDM-1	147	K64-O2a	16 (R)	>16 (R)	ND	>32 (R)	>8 (R)	>16 (R)	>2 (R)	0.5 (S)	≤1 (S)	>8 (R)	>8 (R)	32 (R)	>64 (R)	ND	ND	>32 (R)	ND	0.25 (S)	0.094 (S)
Kpn8	12/12/2023	Urinary tract	NDM-1	147	K64-O2a	16 (R)	>16 (R)	ND	>32 (R)	>8 (R)	>16 (R)	>2 (R)	0.5 (S)	>8 (R)	>8 (R)	>8 (R)	>32 (R)	>64 (R)	ND	ND	>32 (R)	ND	2 (S)	0.38 (S)
Kpn9	05/01/2024	Blood	NDM-1	512	unk-O2afg	32 (R)	>16 (R)	ND	>32 (R)	>8 (R)	>16 (R)	>2 (R)	0.5 (S)	>8 (R)	>8 (R)	>8 (R)	>32 (R)	>64 (R)	ND	ND	>32 (R)	ND	2 (S)	0.5 (S)
Kpn10	10/01/2024	Wound swab	NDM-1, KPC-3	512	unk-O2afg	32 (R)	>16 (R)	ND	>32 (R)	>8 (R)	>16 (R)	>2 (R)	0.5 (S)	>8 (R)	>8 (R)	>8 (R)	>32 (R)	>64 (R)	ND	ND	>32 (R)	ND	2 (S)	0.75 (S)
Kpn11	11/01/2024	Urinary tract	NDM-1, KPC-3	9095	unk-O2afg	32 (R)	>16 (R)	ND	>32 (R)	>8 (R)	>16 (R)	>2 (R)	2 (S)	>8 (R)	>8 (R)	>8 (R)	32 (R)	>64 (R)	ND	ND	>32 (R)	ND	1 (S)	0.25 (S)
Kpn12	17/01/2024	Rectal swab	NDM-1	147	K64-O2a	16 (R)	>16 (R)	>32 (R)	>32 (R)	>8 (R)	>16 (R)	>2 (R)	0.5 (S)	≤1 (S)	>8 (R)	>8 (R)	>32 (R)	>64 (R)	160 (R)	>8 (R)	>32 (R)	4	2 (S)	0.25 (S)
Kpn13	18/01/2024	Rectal swab	NDM-1, KPC-3	512	unk-O2afg	32 (R)	>16 (R)	>32 (R)	>32 (R)	>8 (R)	>16 (R)	>2 (R)	0.5 (S)	>8 (R)	>8 (R)	>8 (R)	>32 (R)	>64 (R)	>160 (R)	>8 (R)	>32 (R)	2	2 (S)	0.5 (S)
Kpn3	10/04/2024	Rectal swab	NDM-1	147	K64-O2a	16 (R)	>16 (R)	>32 (R)	>32 (R)	>8 (R)	>16 (R)	>2 (R)	0.5 (S)	>8 (R)	>8 (R)	>8 (R)	>32 (R)	>64 (R)	>160 (R)	>8 (R)	>32 (R)	4	1 (S)	0.38 (S)
Kpn1	12/04/2024	Rectal swab	NDM-1	395	K2-O1ab	32 (R)	>16 (R)	>32 (R)	>32 (R)	>8 (R)	>16 (R)	>2 (R)	0.5 (S)	>8 (R)	>8 (R)	>8 (R)	32 (R)	>64 (R)	>160 (R)	>8 (R)	>32 (R)	1	4 (R)	0.125 (S)
Kpn2	12/04/2024	Blood	NDM-1	147	K64-O2a	ND	>16 (R)	ND	>32 (R)	>8 (R)	>16 (R)	>2 (R)	0.5 (S)	≤1 (S)	>8 (R)	>8 (R)	32 (R)	>64 (R)	ND	ND	>32 (R)	ND	0.5 (S)	0.19 (S)
Kpn5	19/04/2024	Rectal swab	NDM-1	147	K64-O2a	16 (R)	>16 (R)	>32 (R)	>32 (R)	>8 (R)	>16 (R)	>2 (R)	0.5 (S)	≤1 (S)	>8 (R)	8 (I)	32 (R)	>64 (R)	>160 (R)	8 (R)	>32 (R)	ND	1 (S)	0.125 (S)
Kpn4	09/05/2024	Rectal swab	NDM-1, KPC-3	512	unk-O2afg	32 (R)	>16 (R)	>32 (R)	>32 (R)	>8 (R)	>16 (R)	>2 (R)	0.5 (S)	>8 (R)	>8 (R)	>8 (R)	32 (R)	>64 (R)	>160 (R)	>8 (R)	>32 (R)	4	2 (S)	0.38 (S)

^*a*^ The table reports the MIC values obtained with antimicrobial susceptibility tests (ASTs).

^*b*^ S, susceptible; R, resistant; I, intermediate; ND, not determined. The antimicrobial agents tested were as follows: AMK, amikacin; FEP, cefepime; CTX, cefotaxime; CAZ, ceftazidime; CZA, ceftazidime-avibactam; C/T, ceftolozane-tazobactam; CIP, ciprofloxacin; CST, colistin; GEN, gentamicin; IMR, imipenem-relebactam; MEM, meropenem; MEV, meropenem-vaborbactam; TZP, piperacillin-tazobactam; AMC, amoxicillin-clavulanic acid; TMP/SMZ, trimethoprim-sulfamethoxazole; TOB, tobramycin; TGC, tigecycline; ATM, aztreonam; FDC, cefiderocol; AZA, aztreonam/avibactam.

^*c*^ MIC values obtained using the VITEK 2 system.

^*d*^ Carbapenemases were detected with immunochromatographic tests, while the specific variant was revealed by WGS analysis.

^*e*^ Resistance to colistin was confirmed using Sensititre.

^*f*^ Cefiderocol MIC values obtained using ComASP.

^*g*^ Tigecycline MICs values were interpreted according to EUCAST Guidance Document on tigecycline dosing.

^*h*^ Gray shading highlights the distinction between sample characteristic descriptions and MIC values.

Some notable differences in antibiotic susceptibility were observed both between *K. pneumoniae* and *E. coli* isolates and within isolates of the same species. All *E. coli* isolates harboring either NDM-5 or VIM-1 exhibited susceptibility to colistin, tigecycline, and cefiderocol. Compared to other STs, the ST1485 isolate was unique in its susceptibility to gentamicin, aztreonam, and meropenem-vaborbactam. Unlike ST648 isolates, which were susceptible to trimethoprim-sulfamethoxazole, ST405 and ST1485 isolates displayed a high level of resistance to this antibiotic combination. Similar to *E. coli*, *K. pneumoniae* strains carrying an MBL, alone or with KPC-3, demonstrated broad resistance to β-lactams and fluoroquinolones. Of note, all isolates resistant to colistin were VIM-carrying *K. pneumoniae*, belonging to ST225 and ST392, while NDM-1-positive Kpn11 showed reduced susceptibility (MIC = 2 mg/L). Although cefiderocol susceptibility was widely reported, nearly half of isolates exhibited an MIC value of 2 mg/L, which is extremely close to the EUCAST breakpoint (resistant phenotype with MIC >2 mg/L), indicating a significantly reduced susceptibility margin.

### Genomic analysis: sequence type, resistance, and virulence

Based on MLST analysis, a heterogeneous ST distribution was observed. In particular, among the 17 *K*. *pneumoniae* strains, ST147 was the most prevalent ST detected in our investigation (eight isolates), followed by ST512 (four isolates), ST225 (two isolates), ST392 (one isolate), and ST395 (one isolate). Furthermore, one isolate belonged to an ST not previously identified. Among the five *E. coli* isolates, three isolates were assigned to ST648, while the remaining two isolates belonged to ST1485 and ST405, respectively. Furthermore, plasmid content was investigated in each isolate. Among *K. pneumoniae* isolates, the most prevalent plasmid Inc type is IncFIB. In *E. coli* isolates, the most frequently observed Inc types appear to be IncFIB(AP001918) and IncFII(pRSB107), often co-occurring with other plasmids like Col-types (File S1).

**Fig 1:**
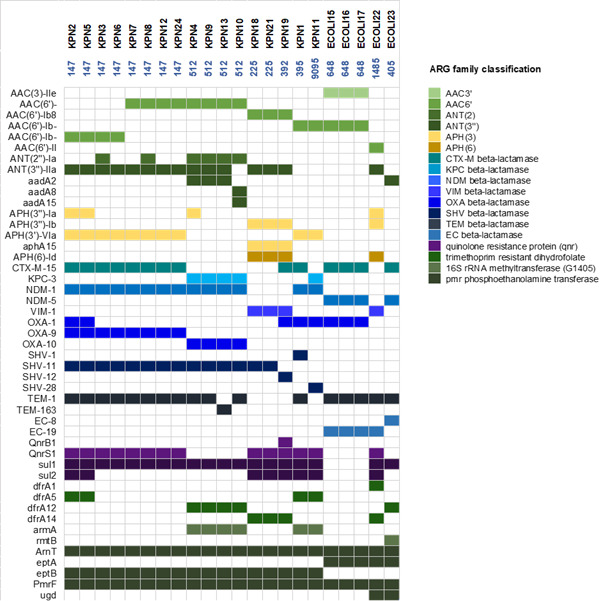
ARG distribution among the isolates analyzed. Each column represents a single bacterial isolate, with its ST indicated at the top, and each row corresponds to a specific resistance gene. A colored cell indicates the presence of that particular gene in the corresponding isolate. The identified ARGs have been classified based on their respective gene family.

Genomic characterization of the isolates revealed a complex resistance landscape, characterized by the occurrence of porins, efflux pumps, and enzyme variants responsible for their altered functional profiles (File S2). The ARGs responsible for the observed resistance to the antibiotics tested in this study are summarized in Fig. 1.

WGS showed that genes encoding various β-lactamases appear to be highly prevalent and that all isolates carried MBL genes located on plasmids (File S3). Among MBLs, NDM-1 was detected in all NDM-producing *K. pneumoniae*, while NDM-5 was limited to *E. coli* ST648 and ST405. In contrast, VIM-1 was found both in *E. coli* (one ST1485) and *K. pneumoniae* (two ST225, one ST392) isolates. Of note, five *K*. *pneumoniae* isolates, belonging to ST512 and ST9095, were characterized by the concomitant presence of NDM-1 and KPC-3. Additionally, we identified other β-lactamases, with the most common being OXA (found in 18/22 isolates), followed by SHV (17/22), TEM (18/22), and CTX-M-15 (14/22). Carbapenemase-encoding *blaKPC* and β-lactamase-encoding *blaEC* were less common. A notable finding was the presence of a remarkably high number of β-lactamase-encoding genes in 11 *K*. *pneumoniae* strains, ranging from five to six per isolate (Fig. 1). Moreover, the co-occurrence of distinct variants of the same β-lactamase (OXA-1 and OXA-9) was also observed. Genes associated with aminoglycoside resistance were found. Specifically, genes encoding aminoglycoside-modifying enzymes (AAC3′, AAC6′, ANT(2), ANT(3′′), APH(3), APH(6)) showed varying and wide distribution among the isolates. Instead, *armA* and *rmtB* appeared to be less common. Among the determinants related to sulfamethoxazole and trimethoprim resistance, *sul* genes (*sul1*, *sul2*) and *dfrA* genes (*dfrA1, dfrA5, dfrA12, dfrA14*) were identified in 19/22 isolates and 13/22 isolates, respectively. Additionally, most isolates exhibited genes related to quinolone resistance, *qnrS1,* either alone or in combination with *qnrB1*. Genes related to polymyxin resistance, including *pmrF* and *arnT*, were detected in all isolates. An *ept* gene (*eptA* or *eptB*), implicated in colistin resistance, was widely distributed, while the *ugd* gene was present in two isolates. Finally, other genes like *catA*, *pmrA*, and *pmrB* exhibited limited distribution in the analyzed isolates. Focusing on the most prevalent STs for each species, we discovered that *K. pneumoniae* ST147 exhibited a wide range of resistance determinants associated with resistance to β-lactams (SHV variants, TEM-1, CTX-M-15, OXA variants), aminoglycosides, fluoroquinolones, and sulfonamides-trimethoprim. *E. coli* ST648 carried multiple genes conferring resistance to aminoglycosides and fluoroquinolones, in addition to NDM-5 and CTX-M-15.

**Fig 2:**
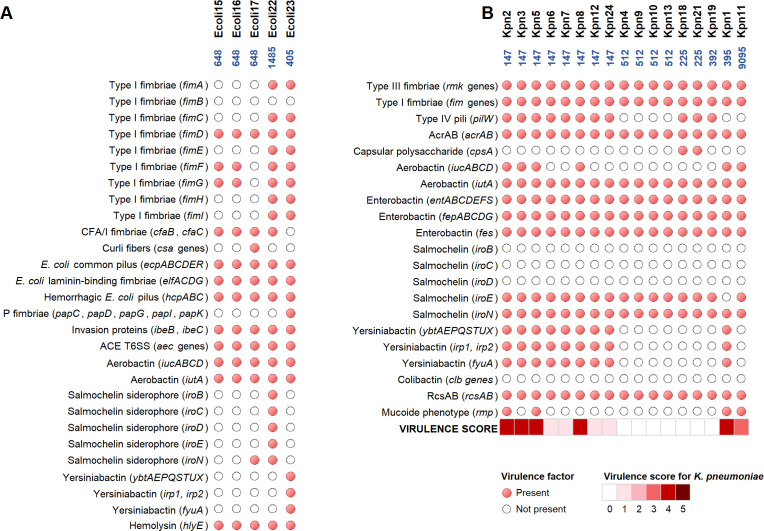
Virulence factor-encoding gene distribution among the *E. coli* (**A**) and *K. pneumoniae* isolates (**B**). Red circles indicate the presence of the gene. Virulence score (0–5) was assigned to *K. pneumoniae* strains according to Kleborate analysis.

Virulome analysis demonstrated a heterogeneous distribution of virulence factor-encoding genes across bacterial species and sequence types, as illustrated in Fig. 2.

Among them, iron uptake systems are of great concern. Particularly, genes for enterobactin (*entABCDEFS*, *fepABCDG*, *fes*) were widely distributed in all *K. pneumoniae* isolates, while aerobactin-encoding genes have a variable distribution, with *iucABCD* operon found in all *E. coli* isolates and in 6 out of 17 *K*. *pneumoniae* isolates, and *iutA* was present in all the analyzed isolates. Moreover, all *K. pneumoniae* isolates and two *E. coli* isolates (Ecoli17 and Ecoli22) carried at least one gene from the salmochelin-encoding *iroBCDEN* operon. Furthermore, yersiniabactin-encoding genes (*ybtAEPQSTUX*, *irp1*, *irp2*, *fyuA*) were detected in *K. pneumoniae* ST147 and ST395 isolates, and in *E. coli* ST405. All *K. pneumoniae* isolates carried *acrAB, fim,* and *rmk* genes, encoding the AcrAB efflux pump, type I and type III fimbriae, respectively. Additionally, ST147 and ST225 isolates also harbored genes for type IV pili (*pilW*) and capsule polysaccharide (*cpsA*). According to Kleborate analysis, virulence scores, ranging from 0 to 5, were assigned to assess virulome content. Specifically, the highest virulence score was exhibited by five ST147 and one ST395, while ST9095 and four ST147 were assigned medium and low virulence scores, respectively. Finally, genes associated with capsule polysaccharide synthesis and hypermucoidy (*rmpA*, *rmpA2*, and *rmpC*) were detected in Kpn1, Kpn2, Kpn5, and Kpn11 strains. In contrast, all *E. coli* isolates harbored additional virulence factors, including genes for type I fimbriae, *E. coli* common pilus (ECP), hemorrhagic *E. coli* common pilus (HCP), hemolysin (*hlyE*), and invasion proteins (*ibeB*, *ibeC*, and *tia*).

### Core-genome SNP analysis

**Fig 3:**
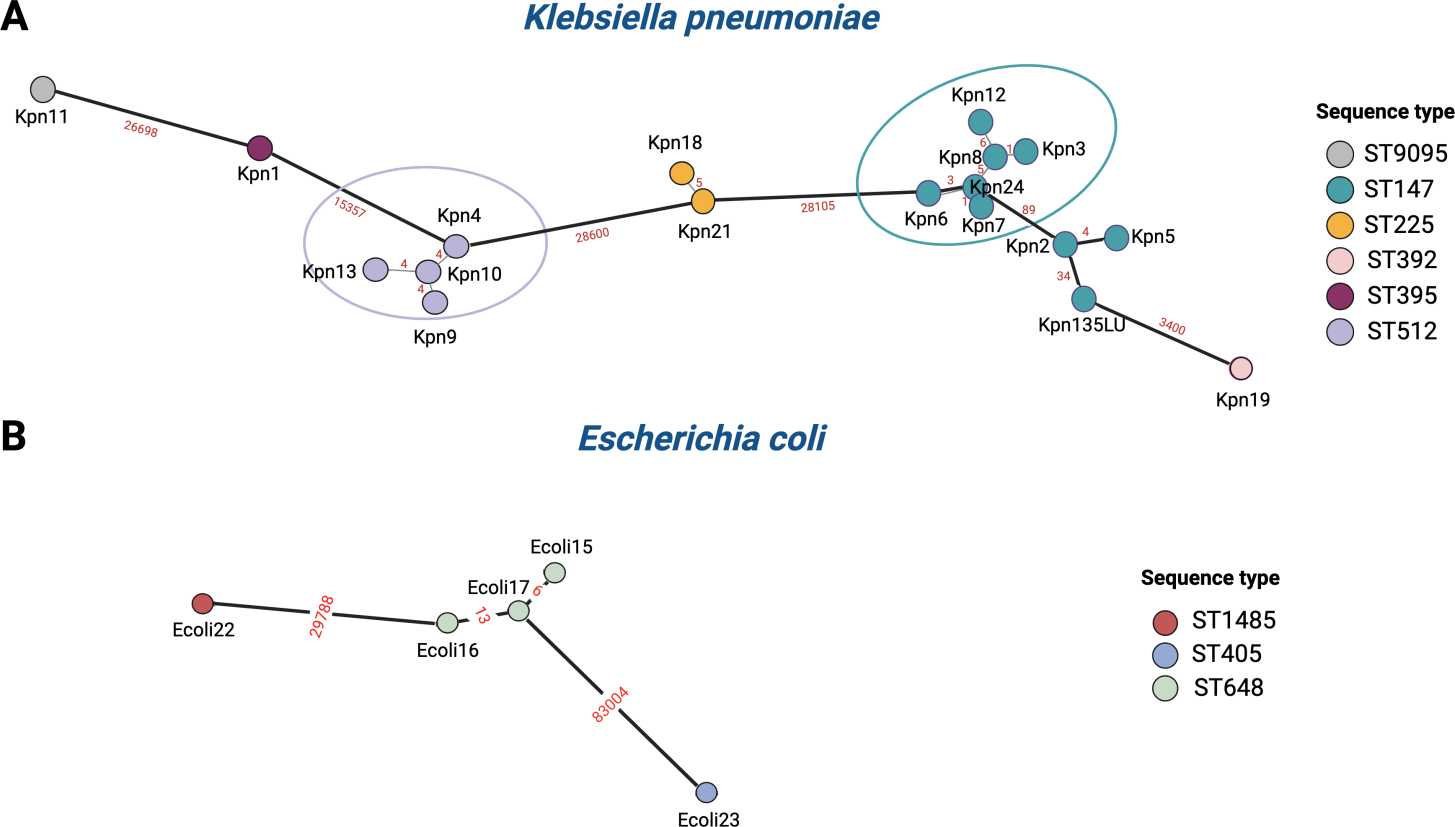
Minimum spanning tree of *K. pneumoniae* (**A**) and *Escherichia coli* (**B**) isolates based on cgSNP analysis. Each node represents a distinct isolate and the colors of the nodes denote different STs. Numbers on lines represent the number of allelic differences between isolates. This figure was created using BioRender (https://www.biorender.com/).

The genetic relationships among the isolates were investigated in detail using core-genome SNP analysis, as shown in Fig. 3. As expected, isolates belonging to different STs generally showed greater genetic diversity. Among *K. pneumoniae* strains, all ST512 isolates demonstrated high genetic similarity, with only four SNPs separating them, suggesting a tightly linked cluster. An additional cluster comprising six isolates, with a median SNP difference of 3, was revealed within the ST147 group. In contrast, Kpn5 and Kpn2, though also ST147, appeared to form a distinct cluster, evidenced by a substantial genetic difference from Kpn24. Furthermore, Kpn2 shows significant genetic relatedness with an NDM-1-producing *K. pneumoniae* clone accountable for an outbreak in Italy (29), named Kpn135LU, diverging by 34 SNPs. Additionally, a high degree of similarity was observed between the two ST225 isolates. Notably, the novel ST9095 isolate displayed considerable genetic diversity compared to the others (Fig. 3A). Regarding *E. coli* ST648, Ecoli15 and Ecoli17 exhibited strong genetic relatedness (6 SNPs different), while Ecoli16 showed greater genetic divergence (13 SNPs different) (Fig. 3B).

## DISCUSSION

MBL-harboring Enterobacterales represent a concerning and growing issue, especially due to their limited treatment options. The epidemiology of carbapenem-resistance mechanisms is shifting, with a progressive spread of NDM-carrying bacteria across Europe, particularly in Italy, where an increasing number of NDM-producing isolates has been reported (9, 10). In this work, conducted in our transplant center, we characterized the clinical, epidemiological, and genomic features of resistance associated with MBL-producing strains isolated from critically ill patients, using phenotypic tests combined with WGS analysis. The most frequently detected MBL was NDM (82% of isolates), with NDM-1 being the predominant variant, followed by NDM-5 found exclusively in *E. coli* isolates. VIM-1 was the only VIM variant detected (18% of isolates). These results align with existing literature that highlights NDM-1 as the most prevalent NDM variant in *Enterobacteriaceae* across European countries, including Italy (30), and NDM-5 variant, exhibiting notable geographic variations, with an endemic presence observed in China (31), and cases sporadically reported in Europe (32, 33). The presence of VIM-producing *Enterobacteriaceae* in our cohort is particularly concerning, as two of these strains were associated with fatal infections. This finding is significant given their reported association with hospital outbreaks (34, 35) and their relatively uncommon occurrence in *Enterobacteriaceae*, compared to *P. aeruginosa* (36). Interestingly, in all the strains, MBL genes had a plasmid localization.

Beyond the expected resistance to cephalosporins, BL/BLI combinations, and carbapenems, due to MBL production, many other antimicrobial agents demonstrated no *in vitro* effectiveness, reducing further therapeutic options. Although MBLs are not able to hydrolyze monobactams, a significant proportion (95%) of the analyzed isolates exhibited aztreonam resistance, likely mediated by class A β-lactamases, including extended-spectrum β-lactamase (ESBLs) and KPCs, which are able to easily inactivate this antibiotic (37). The *in vitro* effectiveness shown by aztreonam-avibactam suggests this antibiotic combination as a promising therapeutic strategy. Resistance to aminoglycosides was also prevalent, with 63% of the isolates resistant to amikacin. Amikacin is often used for MDR Gram-negative bacterial infections treatment, including urinary tract infections (18), particularly when alternative therapeutic agents like cefiderocol are not available. In our study, for instance, 31.8% of patients received an aminoglycoside as part of their empirical antibiotic regimen.

Despite high susceptibility rates being limited to colistin and cefiderocol, we observed the emergence of colistin resistance in all VIM-1-producing *K. pneumoniae* and a relevant number of *K. pneumoniae* isolates showing cefiderocol MICs very close to the EUCAST breakpoint, indicating a reduced susceptibility to this antibiotic. Our results reflect a phenomenon observed in several studies, which report a higher prevalence (42%–59%) of NDMs among cefiderocol-resistant isolates (38, 39). Furthermore, MBLs, such as IMP, VIM, and NDM, can marginally hydrolyze cefiderocol and lead to an increased MIC (40, 41). This is supported by the fact that inhibition of MBL activity using dipicolinic acid prevented the emergence of cefiderocol-resistant mutants (42). On the other hand, we cannot exclude the involvement of other factors not investigated in this study, such as the overexpression of various class A β-lactamases, mainly KPC and SHV, which have been reported to contribute to cefiderocol resistance (43). Although we found only one strain (Kpn1) resistant to cefiderocol, it is important to highlight that cefiderocol resistance has been reported in a large outbreak in Tuscany, Italy, recently (8).

Genotypic analysis revealed a significant presence of concerning high-risk clones (44–46) among the isolates circulating in our hospital. MLST analysis revealed that the most frequent *K. pneumoniae* STs were ST147, known to be responsible for several outbreaks in European countries (Italy, Greece, and, more recently, Spain [47]) and North Africa and India (44, 48), followed by ST512. The most prevalent *E. coli* ST was ST648, described to become a high-risk clonal lineage, alarmingly circulating worldwide (49, 50), and to worsen infection treatment possibilities (51). Besides them, we found other STs, including one not previously described. The substantial heterogeneity in ST distribution strongly suggests multiple independent acquisitions, and no evidence of a defined outbreak was identified during the investigation.

According to the phylogenetic analysis, two significant potential clusters within the *K. pneumoniae* group were present, one comprising all four ST512 isolates and another one consisting of six out of eight ST147 isolates, both showing strong genetic relatedness. Remarkably, one of the ST147 isolates (Kpn2) shows significant genetic relatedness with an NDM-1-producing *K. pneumoniae* clone responsible for a large outbreak in Tuscany, Italy (29). Notably, the significant genetic diversity shown by the novel ST9095 isolate suggests the new ST is unlikely to derive from other detected strains. Our data, especially the intra-ST147 diversity, show that MLST alone may not be sufficient for a detailed epidemiological tracing, while the cgSNP analysis appeared to be crucial.

The resistome analysis revealed a complex landscape of antibiotic resistance mechanisms among the isolates analyzed. Several ARGs appear to be particularly widespread. For instance, genes associated with resistance to β-lactams (e.g., *SHV*, *TEM*, *CTX-M*, *KPC,* and *OXA* members) are prevalent in many *K. pneumoniae* isolates. Among the genes mentioned above, blaSHV-11 encoding β-lactamases has been involved in drug resistance mechanisms (52) through enzymatic inactivation of antimicrobials (53). Similarly, the co-presence of genes conferring resistance to aminoglycosides (*aac*, *ant*, *aph*), fluoroquinolones (*qnr*), sulfonamide (*sul*), and trimethoprim (*dfr*) was often detected. In general, a higher number of genes conferring resistance to aminoglycosides was found in *K. pneumoniae* isolates, compared to *E. coli*.

Of note, among the NDM-producing *K. pneumoniae* (ST147, ST395) and *E. coli* (ST648 and ST404) strains examined in this study, the concomitant presence of ESBL, including CTX-M-15, OXA, SHV, and TEM variants, was observed. Overall, our findings suggest a general correspondence between genomic resistance profiles and phenotypic antibiogram. However, while the absence of resistance genes often predicts susceptibility, the mere presence of genetic determinants is not always sufficient to confer resistance to an antibiotic, and this is probably due to the complex and multifactorial mechanisms underlying antimicrobial resistance profiles. For instance, the molecular mechanisms leading to colistin resistance involve multiple interacting factors, mainly centered on LPS modification (54). One such factor is the plasmid-borne mobile colistin resistance gene (*mcr-1*), which was not detected in isolates analyzed here. Conversely, WGS revealed a widespread diffusion of *pmrF*, *arnT*, and *ept* gene variants, reported to potentially confer polymyxin resistance (55, 56). The low rate of colistin resistance observed in this study could be explained by their different expression levels, which were not tested. Furthermore, other resistance strategies, such as efflux pumps and capsule formation (57), may also be implicated in these isolates’ resistance mechanisms.

All the isolates characterized in this study harbored several virulence factors, with significant clinical implications. Particularly, siderophore systems, involved in bacterial growth and pathogenesis (58, 59), were consistently detected, mainly in *K. pneumoniae* isolates and *E. coli* ST1485 and ST405, which harbored the highest number of virulence factors. All the *E. coli* isolates carried factors involved in colonization and biofilm formation (ECP and HCP) (60), determinants involved in adhesion (laminin-binding fimbriae) (60), proteins with toxic effects (hemolysin), and invasion proteins. In particular, the Ecoli23 strain was also characterized by 39P fimbria playing a role in ExPEC pathogenicity (61). Strikingly, this was the only *E. coli* strain in our study that resulted in patient death, whose infection was identified as the primary cause. The presence of these virulence determinants may explain why 40% (2/5) of the patients infected with MBL-producing *E. coli* developed a subsequent infection following the initial isolation.

All the *K. pneumoniae* isolates were positive for type III and I fimbriae, which facilitate bacterial adhesion (62), the AcrAB efflux pump involved in expelling antibiotics and antimicrobial peptides (63), and *rcsAB*, a regulator of capsule synthesis, as well as siderophores. Furthermore, the regulator of mucoid phenotype, *rmp* (64), was detected in some of the strains. Finally, through a bioinformatic tool, a very high virulence score was assigned to five strains (Kpn1, Kpn2, Kpn3, Kpn5, and Kpn8). Four of these high-virulence isolates (Kpn2, Kpn3, Kpn5, and Kpn8) belong to ST147, well known as a global MDR high-risk clone (44). These isolates were associated with subsequent infections in patients, resulting in two cases of patient death, highlighting the significant clinical impact of such strains on the clinical outcome.

Overall, our findings revealed an alarming co-presence of multidrug resistance and virulence among MBL-producing Enterobacterales, particularly for *K. pneumoniae*. Virulome profiling identified a broad repertoire of virulence factors, including siderophores and fimbriae, with several strains linked to subsequent infections and poor clinical outcomes. Notably, widespread resistance determinants, including those conferring resistance to aminoglycosides, fluoroquinolones, and last-resort agents like colistin and cefiderocol, severely narrow therapeutic options. In addition to colistin-resistant strains, we observed a worrying trend of reduced susceptibility to cefiderocol, which is particularly relevant given its growing role in the treatment of MBL-producing organisms, thus warranting close monitoring.

Although the limitation of the study resides in the relatively small sample size and the monocentric design, our findings offer valuable insights into the genomic and clinical landscape of MBL-producing pathogens in this specific patient population. Bigger and multicenter studies may be useful to better define genetic and virulence-associated factors linked to poor outcomes and ultimately guide earlier, more targeted interventions to improve patient survival.

In conclusion, genomic analyses are crucial for a prompt understanding of epidemiological dynamics and resistance mechanisms. Resistome and virulome information, obtained with WGS, could be used to develop new infection control protocols, able to track hypervirulent MDR strains associated with difficult-to-treat infections and hospital outbreaks.

## Data Availability

The raw Illumina WGS data are available in the NCBI SRA database with the accession number: PRJNA1299079.

## SUPPLEMENTAL MATERIAL

Supplemental file 1 spectrum.02077-25-s0001.xlsx Genomic features of the isolates analyzed.

Supplemental file 2 spectrum.02077-25-s0002.xlsx Complete list of ARGs detected in isolates analyzed.

Supplemental file 3 spectrum.02077-25-s0003.xlsx Plasmid localization of MBL genes.

ASM does not own the copyrights to Supplemental Material that may be linked to, or accessed through, an article. The authors have granted ASM a non-exclusive, world-wide license to publish the Supplemental Material files. Please contact the corresponding author directly for reuse.
